# Case of Rape—Death the Consequence

**Published:** 1851-11

**Authors:** R. S. Bailey

**Affiliations:** Charleston, S. C.


*Case of Rape.—Death the Consequence.* By R. S. Bailey, M. D., Charleston, S. C.—August 10th, 1821.—Was called to visit a negro girl at the plantation of Mrs. LeGay, in Christ Church Parish, distance about eight miles. On seeing the patient, I was informed that she had been ill a week, and supposed to have dysentery, till this morning, when she confessed that violence had been done to her by a negro man, on the 3d inst., and who had since absconded. On examination, I found her extremities cold, pulse scarcely perceptible, continued pain in the hypogastric region, tongue furred, no appetite; her eyes have a glassy appearance; she has made no urine, and passes nothing but a bloody matter, *per ano;* the parts are tender to the touch; excoriation about the anus; on passing the finger *per ano* and vaginam, could only observe a thin partition of cellular membrane, not thicker than the tunicha archnoides. The urethra appears inflamed; on introducing the catheter, a few drops of urine were discharged, followed by a discharge of pus, which besmeared the catheter. Ordered a warm bath, fomentation to abdomen and injection; also, prescribed the following powders:

℞. Pulv. Cinchon,............1 drachm.

Potass Nitrat, ............½ "

Pulv. Opii, ............2 grs.

divided into six powders, one to be taken every three or four hours. She diedsoon after my visit.—*Charleston Medical Journal.*

